# Mesenchymal stem/stromal cells: the therapeutic effects in animal models of acute pulmonary diseases

**DOI:** 10.1186/s12931-020-01373-5

**Published:** 2020-05-11

**Authors:** Sirous Sadeghian Chaleshtori, Mohammad Reza Mokhber Dezfouli, Massoumeh Jabbari Fakhr

**Affiliations:** 1 Department of Internal Medicine, Faculty of Veterinary Medicine, University of Tehran, Tehran, Iran; 2 Institute of Biomedical Research, Faculty of Veterinary Medicine, University of Tehran, Tehran, Iran; 3 Department of Tissue Engineering, Faculty of Medicine, Qom University of Medical Sciences, Qom, Iran

**Keywords:** Mesenchymal stem/stromal cells, Pulmonary diseases, Therapeutic effects

## Abstract

The pulmonary diseases are one of the most important causes of death in the world. The successful therapies in the field of lung diseases are very limited and the medical treatments available are ineffective in many of the lung diseases. Many studies have evaluated the new therapies in the acute pulmonary diseases, and the transplantation of mesenchymal stem/stromal cells (MSCs), which is a branch of cell therapy, has a special place among the new medical techniques. The MSCs are present throughout the body and are thought to play a role in tissue regeneration and inflammation control. In the event of injury, the local MSCs traverse the shortest possible distance from the tissue or blood vessels to reach the affected site. But, there are few undifferentiated cells in the tissues. The exogenous MSCs are used to immunity modify or regenerative treatments in preclinical models of acute pulmonary diseases. Several studies have shown the positive effects of MSCs replacement in the acute lung disorders. The effection mechanism of the MSCs include the differentiation ability and the secretion of paracrine agents such as the anti-inflammatory mediators. Many studies suggest that this treatment method is safe and is probably to be widely used in future clinical trials. This review will describe the therapeutic effects of the MSCs in the experimental models of the acute pulmonary diseases for use as a method of treatment in clinical trials in future.

## Importance of pulmonary diseases

The acute pulmonary diseases in all countries of the world cause irreparable damage and the mortality rate of pulmonary involvement is high worldwide. The reports show a different and widespread mortality rate for the various pulmonary diseases. For example, a systematic review shows that the acute respiratory distress syndrome (ARDS) mortality is 36–44% [1].

With regard to the anatomy and physiology of the lung tissue and successive contact with organic and non-organic substances, despite the existence of multi-faceted defense mechanisms and the advanced intrinsic and acquired immune defense system, this organ is regularly exposed to pathogens that result in damage to the structural cells [2]. On the other hand, large amounts are spent on diagnosis, hospital care, outpatient services, emergency services, drug therapy and self-care for patients with the acute pulmonary diseases, annually.

Stem/stromal cells transplantation is a branch of cell therapy that has a special place among the new medical methods. Many studies suggest that this treatment method is safe and most probably will be widely used in future clinical trials [3]. Today, the MSCs-based cell therapy is a promising method for treatment of acute diseases of the lung. Regarding the nature and the ability of MSCs in secreting the different paracrine agents, such as growth factors, the endothelial and epithelial permeability regulatory factors and the anti-inflammatory cytokines are effective in the treatment of acute pulmonary disorders [4, 5].

## Pathophysiology of pulmonary diseases

A pulmonary disease may be the result of involvement of the respiratory airways, the lung parenchyma or the blood vessels. A combination of these disorders is seen in many pulmonary diseases. The pathogenesis of pulmonary diseases includes damage to the lung capillaries endothelial cells, damage to the alveolar epithelium, followed by accumulation of protein-rich fluids, cellular lesions in the alveolar space and the inflammation of airways that cause resistance to air flow and the lung capacity reduction [6, 7]. At the cellular level of the lungs the invasion of polymorph nuclear leukocytes (PMNs), including the neutrophils and cytokines to the lung capillaries also occurs, then their placement and adhesion to the endothelium, followed by an acute inflammatory reaction. Neutrophil is the first cell that goes into inflammation site and secretes strong antimicrobial agents, including oxidants, proteases, platelet activating factors, leukotrienes and cationic peptides. The alveolar macrophages secrete and active neutrophils are the inflammatory mediators that interrupt the epithelial fluid transfer and production of surfactant by the alveolar type II (ATII) cells [8].

The direct consequence of infection and subsequent inflammation is imbalance in the fluids of the lung capillaries and increased lung permeability, which disturbs the endothelial cells of the capillaries. With the breakdown of these cells, congestion occurs in the lung veins. Initially, additional fluid interstitial lung tissue is taken by the lymph system and returned to the vascular system. Hydrostatic forces change from capillary endothelium damage. Continuous fluid accumulation is affected lymphatic drainage and hydrostatic forces, and excess fluid to the alveolus leads to non-cardiac edema. Damage to the capillary-alveolar barrier changes the active transfer of sodium and leads to impaired clearance of the edema fluid from the alveolar spaces. Pulmonary edema is caused by irregular function of ionic channels in alveolar cells [7, 9].

Also, pathologic permeability of the vessels, the gap in the capillary-alveolar barrier and necrosis of the alveolar type I (ATI) and ATII cells lead to the formation of a hyalin membrane and destroying surfactant, which reduces the pulmonary capacity and disturbs the exchange of respiratory gases causing hypoxia and hypercapnia. Finally, fibroblastic secretions can lead to collagen deposition, fibrosis and worsening of the disease [10]. Many patients develop deep muscle weakness and as a result, respiratory failure occurs and their quality of life decreases.

## The process of pulmonary repair in injury/inflammation

The process of lung repair after injury/inflammation is a complex phenomenon. Endogenous stem/stromal have potency for healing injured epithelium via adaptation their structure and function. The experiment on epithelium repair of airways show that the recovery process of airway epithelium such as differentiation, migration of adjacent epithelial cells to cover the empty region, proliferation of precursor cells to restore cell number and differentiate of cells to restore the function of epithelial cells [11].

Airways epithelium immediately after injury, begins a recovery process to restore integrity to the air-blood barrier. The ATII cells proliferate and differentiate into the ATI cells, which restore the integrity of the epithelial coating and create the osmotic gradient. This osmotic gradient causes the fluid to flow out of the alveoli and into the small vessels and lymph nodes. The anti-inflammatory cytokines disable the harmful neutrophils and then the neutrophils are apoptosis [12].

Active PMNs act to repair lung damage by secreting a range of destructive factors, including elastase neutrophil, metalloprotease, other proteolytic enzymes and oxidants that are effective in improving lung function. Simultaneously, the macrophages and alveolar cells remove protein compounds from the alveoli and improve the lungs [12]. There are also chemokine-dependent migratory macrophages that can contribute in the recovery process by releasing cytokines and apoptosis-inducing molecules [13].

## Challenges facing pulmonary diseases

When the damage to the lung and airway is more than the compensatory power of the restorative mechanisms, treatment should begin immediately. The successful therapies in the field of lung diseases are very limited and the drug treatments available are ineffective in many lung diseases [14]. Many studies have evaluated the new therapies in pulmonary diseases, which focus on manipulating inflammatory pathways at various levels, reducing vascular disorders, reducing lung oxidative damage and optimizing lung tissue repair. The current therapies are based on mechanical ventilation and oxygen membranes, neuromuscular blocking agents, proper fluid delivery, oxidants, surfactants, corticosteroids, antimicrobial treatment and use of steroids and beta-agonists [1, 15, 16]. The diseases of interstitial lung tissue causes gradual fibrosis and dyspnea, and do not respond to existing treatments [14]. Despite the advancement of therapeutic approaches, lung transplantation remains the only therapeutic strategy for many patients, which it is not feasible due to lack of donor tissue or shortage of suitable donor. Severe lung donor shortage causes many patients on the waiting list to die before transplantation [2]. In addition, lung transplantation has poor potential in treating patients, and the transplanted lungs have a lifetime low. The researches show that within 5 years after transplantation, about 50% and during 10 years, 75% of the transplanted patients lose their lives. On the other hand, in successful transplantations, the lifelong need for immunosuppressive drugs will cause many problems and costs for the patient [17]. Therefore, the development of preventive approaches and the new therapies are important to reduce pulmonary diseases and improve the quality of life.

Today, the treatment of diseases has shifted to cell therapy, tissue engineering and gene therapy. Recent findings suggest that embryonic stem cells and stem/stromal cells isolated from adult tissues may play a role in restoration and repair of the damaged tissues [3, 18]. The most common clinical application of stem/stromal cells is related to the bone and cartilage tissues, the urinary tract, the nervous system and cancers. In the field of lung and airways, due to the specific physiological complexities, little research has been done on the treatment with stem/stromal cells. With regard to the high proliferative power and the ability to multi-lineage differentiate MSCs and, in particular, because of their significant role in the effects of immune modification [19, 20], these cells can be used as a practical tool for cell therapy purposes for treating a large number of degenerative diseases and congenital disorders.

## Stem/stromal cells and precursor cells

The stem/stromal cells are defined as non-specific cells that have significant potential for conversion into a variety of cell types. These cells can restore the place of damaged cells with a system of internal restoration [21]. Most organs of the body have a small reservoir of stem/stromal cells that is capable of proliferation, differentiation into precursor cells, replace old cells and repair minor injuries [22]. The potential sources of precursor cells are identified for epithelium of the lung and airways and are divided into two groups: the endogenous and exogenous stem/stromal cells.

### Endogenous stem/stromal cells

The undifferentiated endogenous stem/stromal cells play roles in the repair and maintenance of all body tissues. There are few undifferentiated cells in the tissues that are often converted into the differentiated cells in the tissue [23]. The lung is a complex organ with more than 40 different and distinct cell types, of which there are different populations of ancestral cells in different anatomical regions along the lung bronchial tree [17].

The researches have shown that lung cells are mature and differentiated and have different phenotypes. In addition to the fundamental role of lung precursor and endogenous cells in repairing the damage, reversible phenotype in the epithelial cells and mucus plays an important role in repairing the damage [24]. Although improvements have been made in clarifying the identity and role of the lung airways precursor cells in mice, the role of this cell population remains uncertain and there is little information in humans and the other animal models. Types of endogenous stem/stromal cells in the lung and airways include: basal cells in the trachea and the upper parts of the bronchi, clara cells in the lower parts of the airways, bronchoalveolar duct junction (BADJ) at the intersection of the bronchioles with the alveoli and ATII cells in the alveoli [17].

### Exogenous stem/stromal cells

The ability of the lung endogenous precursor cells to repair, which is often considered as a natural regeneration capacity, decreases with age, and although damage can be quickly and completely recovered by precursor cells, these cells are not usually enough to compensate widespread injuries. The purpose of transplantation of exogenous stem/stromal cells (such as embryonic, adults, etc.) is to replace damaged cells or dead cells. The transplantation of autologous or allogenic cells in models of animal has indicated that cells can be replaced in the lung and differentiated into the adult epithelium phenotypes and as a result, increased cellular response to damage [25]. Exogenous stem/stromal cell-based therapies, including MSCs, has created a new approach to acute diseases of the lung.

## Mesenchymal stem/stromal cells

The MSCs are present throughout the body and are thought to play a role in tissue regeneration and inflammation control. In the event of injury, the local MSCs traverse the shortest possible distance from the tissue or blood vessels to reach the affected site. The isolated MSCs from adipose tissue, blood or bone marrow are released in response to inflammation and absorbed through the lymph nodes and blood vessels by the chemokine-dependent mechanism [26]. The MSCs are cells with high proliferation and self-healing. The differentiation power of these cells in vivo and in vitro has led them to be considered as a good tool for regenerative medicine and tissue engineering. With regard to the high proliferation and the ability MSCs differentiation and also because their prominent role in the effects of immune modulation, the ability to migrate to injury sites, the secretion of growth factors, anti-inflammatory cytokines, extracellular vesicles as well as action through differentiation and cell-cell junction [19, 20, 27, 28] can be used as an applied tool for the purpose of cell therapy for the treatment of congenital disorders, degenerative diseases, autoimmune diseases and also as efficient carriers to deliver treatment to specific areas [15, 29].

The studies have shown that cell therapy is more effective in the acute phase of pulmonary diseases such as obstructive pulmonary disease (COPD), idiopathic pulmonary fibrosis (IPF), bronchopulmonary dysplasia (BPD), cystic fibrosis (CF) and ARDS than other conflicts [17]. The recent studies have displayed the effects of paracrine in these cells, such as angiogenesis stimulation, adjustment of inflammatory conditions and immune responses in animal models of pulmonary diseases [27, 28]. A new therapeutic strategy using MSCs causes the significant decline in the acute pulmonary inflammation induced by Lipopolysacaride (LPS) in the animal models of mice [27, 28, 30], rabbit [5] and sheep [31].

A part of the transplanted MSCs in pulmonary diseases migrates to the site of injury and inflammation and creates beneficial effects. The other part may affect the other cells by tissue restorative mediators or immune modulation. The MSCs are able to express a wide range of the chemokine receptors and migrate based on the chemokine slope. Most chemokines have an effect on tumor necrosis factor-α (TNF-α), and MSCs mobilization and their migration to damaged tissues depends on the systemic and topical inflammation amount [32]. The duration of cell culturing and the amount of cell culture have a significant effect on the morphology, differentiation, survival and migration characteristics of the MSCs [33]. The MSCs immediately after isolation have the ability to migrate more quickly than the cultivated MSCs [32, 34]. The different preparation conditions of MSCs make a difference in the cell phenotype and the expression of the migratory receptors, and this may lead to differences in their therapeutic effects [26]. Therefore, culture condition is a powerful and effective tool in the effects of cellular therapies.

## Therapeutic function MSCs

The exogenous MSCs are used to immunity modify or regenerative treatments in preclinical models of various diseases. Several studies have shown the positive effects of MSCs replacement in the acute lung disorders. The MSCs migration also depends on transplantation route. Most studies have used intravenous route [30, 35–39] and a large portion of the MSCs is trapped in the lungs during the first pass [40]. Escape from the lung trap may improve the survival of the MSCs and affect cell distribution and its therapeutic effects after transplantation. In the arterial infusion, there is more certainty about the MSCs reaching target organ. After 24 h, the MSCs move to the other organs, especially the liver and the spleen, and appear in the injured tissue [26].

In inflammatory conditions, the MSCs express Indolearnine 2, 3-dioxygenase (IDO), which plays an important role in the production of regulatory T cells [41]. The MSCs induce regulatory T cells by increasing the Interlukine-2 (IL-2) level, this occurs when active T cells are inhibited by the MSCs [42]. In addition, after the MSCs transplantation, monocytes as a very important mediator cell become the macrophages of type II. The type II macrophages, produce Interlukine-2 (IL-2) (IL-10) and chemokine (C-C motif) ligand 18 (CCL18), which result in the induction of regulatory T cells [26]. Overall, these studies indicate the effects of MSCs on the immune system in acute pulmonary injury in the models of mice and human, and provide important pre-clinical information for clinical trials of the MSCs in the pulmonary diseases.

Different mechanisms of paracrine, including the release of anti-inflammatory mediators such as IL-10, prostaglandin E2 (PGE2) [43], angiopoietin-1 [44] and keratinocyte growth factor (KGF) [45], have been proposed to justify the therapeutic effects of the MSCs. Transplantation of the MSCs results in a rapid response that may subsequently affect the other cells. The systemic nature of these responses represents that even the MSCs in the lung after transplantation can spread their effects to other sites in the body, and this may be the basis of the mechanism of treatment with the MSCs [26]. Considering all of the published information, it should be admitted that there is still little information on how the MSCs therapeutic effects work. Therefore, further studies are needed to explore the fate of the MSCs and their functional characteristics after the transplantation to allow clinicians to use the MSCs clinically.

## MSC therapy in the model of pulmonary diseases

Transplantation of various types of the ancestral cells, including the mononuclear cells isolated from bone marrow, the endothelial progenitor cells, the dental pulp stem cells, the amniotic fluid cells, the hematopoietic cells, etc. have been effective in the different clinical models of the acute pulmonary diseases such as acute lung injury (ALI)/ARDS [15, 46].. However, the available clinical data on the effects of the isolated MSCs from bone marrow is more than the other cells. The various reports indicate the efficacy of stem/stromal cell-based therapies in the significant reduction of the acute pulmonary inflammation induced by LPS in mice [28, 30, 47, 48], rats [48–50], rabbit [5] and sheep [31], the pulmonary inflamma induced by oleic acid in rats [51] and pig [37], the lung inflammation caused by inhalation of smoke in sheep [36] and the inflammation of lung due to H9N2 avian influenza in mice [52] (Table 1).

**Table 1:** The model of acute lung diseases in the various animal species and treatment using the MSCs

	The method of inflammation	The causative agent of inflammation	Animal species	The method of cell transplantation	Dose of the cell	Source of the cell	Type of the cell
[30]	Intratracheal	LPS	Mice	Intravenous	2.5 × 10^5^	Bone marrow of mice	MSCs
[53]	Intrperitoneal	LPS	Mice	Intravenous	5 × 10^5^	Bone marrow of mice	MSCs
[28]	Intratracheal	LPS	Mice	Intratracheal	7.5 × 10^5^	Bone marrow of mice	MSCs
[54]	Intratracheal	E.coli	Mice	Intratracheal	10^6^	Bone marrow of humman	MSCs
[49, 55]	Intravenous	LPS	Rat	Intravenous	10^6^	Bone marrow of rat	MSCs
[56]	Intratracheal	LPS	Mice	Intratracheal	10^6^	Umbilical cord of humman	MSCs
[57]	Intratracheal	E.coli	Mice	Intratracheal	10^5^	Umbilical cord of humman	MSCs
[58]	Aspiration	LPS	Mice	Aspiration	5 × 10^5^	Bone marrow of humman	MSCs
[50]	Intrperitoneal	LPS	Mice	Intravenous	5 × 10^5^	Umbilical cord of humman	MSCs
[59]	Intratracheal	E.coli	Mice	Intratracheal	7.5 × 10^5^	Bone marrow of mice	MSCs
[60]	Intratracheal	LPS	Rat	Intrapleural	10^6^	Bone marrow of rat	MSCs
[51]	Intravenous	Oleic acid	Rat	Intravenous	2 × 10^5^	Bone marrow of rat	MSCs
[61]	Intratracheal	LPS	Mice	Intravenous	5 × 10^5^	Bone marrow of mice	MSCs
[38]	Intratracheal	LPS	Mice	Intratracheal	2.5 × 10^5^	Bone marrow of mice	MSCs
[62]	Intratracheal or Intrperitoneal	LPS	Mice	Intravenous	10^5^	Bone marrow of mice	MSCs
[63]	Ventilation	Ventilator	Rat	Intratracheal or Intravenous	4 × 10^5^	Bone marrow of rat	MSCs
[64, 65]	Intranasal	LPS	Mice	Intravenous	10^6^	Adipose tissue of humman	MSCs
[66]	Intratracheal	LPS	Mice	Intravenous	5 × 10^5^	Bone marrow of mice	MSCs
[67]	Intratracheal	E.coli	Mice	Intravenous	5 × 10^5^	Bone marrow of mice	MSCs
[68]	Intrperitoneal	LPS	Mice	Intravenous	5 × 10^5^	Bone marrow of mice	MSCs
[36]	Intratracheal	Smoke	Sheep	Intravenous	10 × 10^6^	Bone marrow of humman	MSCs
[69]	Intratracheal	LPS	Mice	Intravenous	5 × 10^5^	Bone marrow of mice	MSCs
[70]	Intrperitoneal	LPS	Mice	Intravenous	5 × 10^5^	Bone marrow of mice	MSCs
[71]	Intratracheal	LPS	Mice	Intratracheal	7.5 × 10^5^	Bone marrow and embryonic of human	MSCs
[72]	Intratracheal	E.Coli	Rat	Intratracheal	2 × 10^6^ 5 × 10^6^ 1 × 10^7^ 2 × 10^7^	Bone marrow of human	MSCs
[52]	Intranasal	H9N2 avian influenza virus	Mice	Intravenous	10^5^	Bone marrow of mice	MSCs
[48]	Aspiration	Stomach contents	Rat	Intravenous	5 × 10^6^	Bone marrow of mice	MSCs
[27]	Intratracheal	LPS	Mice	Intravenous	0.5 × 10^6^	Umbilical cord of human	MSCs
[37]	Intravenous	Oleic acid	Pig	Intravenous	2 × 10^6^	Bone marrow of human	MSCs
[35, 73]	Intratracheal	LPS	Mice	Intravenous	10^6^	Menstrual blood	MSCs
[74]	Intratracheal	LPS	Mice	retro-orbital injection	5 × 10^5^	Adipose tissue of mice	MSCs
[31]	Intratracheal	LPS	Sheep	Intratracheal	5 × 10^7^	Bone marrow of sheep	MSCs
[5]	Intratracheal	LPS	Rabbit	Intratracheal	10^7^	Bone marrow of rabbit	MSCs

Johnson et al. stated that cell therapy was commonly performed before or at the time of infection in the designed models to evaluate the effect of stem/stromal cells on damage and this is not suitable for most septic patients [75]. The results also show that the experimental model of the acute pulmonary inflammation peaks after infusion of infectious agents at 24–48 h. Accordingly, in a study by Mokhber Dezfouli et al., transplantation of stem/stromal cell was performed 24 h after inflammation with maximal inflammatory symptoms, in contrast to most of the studies that have performed stem/stromal cell transplantation between 30 min and 4 h after inflammation. Their results indicated the significant effects of MSCs on reducing inflammation and pulmonary edema, and improvement in clinical symptoms compared with control group [5].

Although both intrapulmonary and systemic transplantation of the isolated MSCs from bone marrow lead to decrement of mortality and inflammation and improvement of alveolar fluid clearance despite the minimal transplantation of stem/stromal cells in the lung [76], the researchers found intrapulmonary transplantation of the human MSCs in the lung inflammation caused by *Escherichia coli* (E.coli) in rats [72], bone marrow mesenchymal stem/stromal cells (BM-MSCs) in the ARDS experimental model in rabbits [5] and BM-MSCs in the ARDS experimental model in sheep [31] is superior to intravenous transplantation.

## The effects of MSC therapy on clinical and paraclinical symptoms

The acute pulmonary diseases cause changes in the clinical symptoms including tachycardia, tachypnea, hyperthermia, abnormal lung sounds, nasal discharge, coughing, reduced appetite, etc., and the various studies show the MSCs therapy can prevent significant changes in clinical symptoms in rabbits [5] and sheep [31]. They represented transplantation of BM-MSCs modulates the clinical sings (heart rate, respiratory rate, body temprature, appetite, physical condition and etc.) in the model of ARDS caused by LPS E.coli [5].

Simonson et al., investigated the effects of MSCs in two patients with ARDS who were resistant to treatment; breathing quality, hemodynamic conditions and failure of the other organs (liver and kidney) were recovered. Their results showed the MSCs transplantation as infusion in human causes reducion in the amount of the inflammatory cells and factors in bronchoalveolar lavage (BAL) and plasma and increases arterial oxygen pressure. In parallel, there was a decrease in various pulmonary and systemic factors, including apoptosis of the epithelial cells, alveolar-capillary fluid leakage, and pro-inflammatory cytokines and chemokines [77].

Zhou et al., investigated the effects of BM-MSCs in the lung injury induced by aspiration of the mouse gastric contents. In this study, the green fluorescent protein (GFP)-positive cells were administered via the tail vein. They displayed the decrement of pulmonary edema and inflammation in pathology, increase of partial pressure of arterial oxygen, the decline of protein level and the total number of cells and neutrophils in BAL, and the reduction of TNF and cytokines caused by the neutrophilic activity [48].

Also, Pedrazza et al., showed the use of adipose tissue-derived MSCs in the model of LPS-induced ALI in mice caused the reduction of lung inflammation, oxidative damage and the neutrophil extracellular traps (NETs) release and a remarkable increase in the survival [74].

McIntyre et al., investigated 54 previous studies using meta-analysis, their report showed the treatment with the MSCs significantly reduced the mortality rate (95%, the confidence interval 0.18–0.34). The report revealed that the deaths have reduced considering all the effective factors including the sex and model species of the study, the source of the cell, the preparation, the method of MSCs transplantation and the clinical conditions of the acute pulmonary injury model. Also, the results of review have shown that the MSCs affect on the wide range of animal models and the experimental conditions. The surveys show that this type of treatment can be used for the many different types of lung injury in the future [78].

## The effects of MSC therapy on inflammation factors in BAL and blood

Most studies have demonstrated that direct damage to cytokines involved in the inflammatory cascades contributes to lung injury [52]. In different studies it has been shown that the MSCs are able to reduce the BAL neutrophils, which are one of the most important characteristics of acute lung inflammation [30] and macrophages [63] and, on the other hand, inhibit activation and proliferation of immune cells by secreting a deterrent factor [79, 80]. The assesment of the BAL fluids following the MSCs therapy in LPS-induced inflammation in mice has shown that the amount of inflammatory cells (especially neutrophils), cytokines (TNF-α, interferon-γ (IFN-γ) and IL6), as well as total protein, albumin and immunoglobulin M (IgM) levels is reduced [30]. The other studies confirmed the effects of the BM-MSCs administration on the decline of pro-inflammatory cytokines levels of the INF-γ, IL-1β, IL-6, TNF-α and macrophage inhibitory protein-2 (MIP-2) and increase of anti-inflammatory cytokine level of the IL-10 in plasma and BAL. This despite the low level of stem/stromal cells transplantation in the lung, occurs in response to cellular contacts and soluble factors [28, 59]. In the study of Mokhber Dezfouli et al., by evaluating cellular content and cytokines concentration of plasma and BAL, the MSCs therapeutic efficacy was investigated in ARDS-induced model by LPS in rabbits, which indicated the significant effects in modulating cellular content and cytokines concentration of BAL and Plasma. Their results displayed a decrease in the plasma concentration of the TNF-α and IL-6 and an increase in the plasma concentration of the IL-10 following cell therapy with BM-MSCs in rabbits [5].

Intravenous tranplantation of MSCs causes the systemic and local decline of inflammatory cells. It has been shown that serum levels of IFN-γ, IL-1β, and MIP are reduced following intravenous transplantation of BM-MSCs in mice [53]. Li et al. demonsterated that intravenous use of MSCs in the treatment of acute pulmonary inflammation induced by H9N2 avian influenza in mice caused a significant reduction in plasma and BAL inflammatory factors (IL-1α, IL-6, TNF-α and IFN-γ) and a the corresponding increase in anti-inflammatory cytokine (IL-10), 3 days after cell therapy. The researchers stated that this treatment method reduces the lung permeability and the concentration of the alveolar fluid proteins [52, 81].

The undifferentiated MSCs have the anti-inflammatory effects and are suitable for the improvement of LPS-induced ALI in mice [59]. The another study has shown that the administration of embryonic-derived MSCs, similar to BM-MSC, has reduced the endotoxin-induced inflammatory response, but based on the findings of gene expression, the embryonic-derived MSCs do not have the protective effect on the pulmonary edema and the protein leakage [71]. On the other hand, the researchers found that the menstrual blood-derived MSCs help to improve the permeability of the lung capillaries and reduce tissue damage and also, their beneficial effects are evident by inhibition of IL-1 and increased IL-10 in BAL and increasing the expression of the nuclear antigen and reducing the expression of caspase-3 [35]. In another study, the intravenous transplantation of adipose tissue-derived MSCs in the model of endotoxin-induced ALI in mice lead to a significant decline in protein content, neutrophil counts, and cytokines of pro-inflammatory (TNF-α, IL-6, and MIP-2) in BAL [64].

The human umbilical cord mesenchymal stem/Stromal cells (UC-MSCs) intravenous transplantation in the acute pulmonary inflammation induced by LPS in mice prevented the inflammatory response of macrophages and increased expression of IL-10 [27]. The findings of this study showed that treatment with the UC-MSCs occurs by the secretion of paracrine agents, especially PGE2, and the factors such as IL-6 and IL-13. After transplantation of the UC-MSCs, the pathological lesions decreased and the inflammatory response including the activity of lung myeloproxidase, the amount of protein, neutrophil and expression of the various inflammatory cytokines decreased in BAL 72 h after transplantation. PGE2 is a paracrine agent that can affect macrophage responses and reduce inflammation. The cell therapy causes the induction decrement of inflammatory mediators, chemokines, the activity of mast cells and nuclear factor kappa B (NF-κB) and regulating in the pathway of oxidative stress formation [27]. Additionally, UC-MSCs can produce a high level of the other paracrine agents such as granulocyte macrophage colony stimulating factor (GM-CSF), granulocyte colony stimulating factor (G-CSF), IL-6 and IL-13 in BAL. These factors play a key role in host defense system, proliferation and differentiation of granulocytes and mononuclear phagocytes. In response to pulmonary infections, GM-CSF can increase the number of alveolar exudative macrophages isolated from monocytes to improve host defense [27].

Recently, it has been shown that intravenous transplantation of the MSCs produces a systemic inflammatory response within a few hours after the transplantation [82]. The induction of regulatory cells may be affected by this systemic inflammatory response or by the tissue MSCs or the immune cells. The phagocytosis potential of macrophages and antimicrobial peptides increases following the cell therapy in the aveoli [52]. The active neutrophils and macrophages produce the oxygen free radicals that play an important role in the inflammatory pathways and lead to the cellular damage [1]. The lymphocytes and the group of macrophages produce the macrophage modulator cytokine (IL-10) which is an anti-inflammatory cytokine and the number of macrophages and neutrophil infiltration are reduced and modulate pro-inflammatory cytokines production and function [83]. Also, based on the results obtained from the plasma evaluation in the MSCs intrapulmonary transplantation in rabbits, it has been determined that the cell therapy has a positive effect on the immune system. The researchers indicated, the BM-MSCs in the ARDS model in rabbits resulted in the decrease in pro-inflammatory cytokines and the increase in anti-inflammatory cytokines in the plasma, similar to BAL, which shows a progressive trend [5], the BM-MSCs transplantation in the ALI model in mice caused the tissue inhibitor expression of metalloproteinase-1 to decline and the increment expression of metalloproteinase-8 [62] and the levels of myeloperoxidase activity and malondialdehyde reduction and the anti-oxidant enzyme activities to increase [67]. In the another study, a significant decrement in the influx of inflammatory cells (the total white blood cells, neutrophils, lymphocytes, and monocytes) and cytokines/chemokines (MIP-2 and TNF-α) has been confirmed following the intrapulmonary transplantation of the MSCs after endotoxin administration in mice [71]. These results represent the function of the MSCs to improve the immune system and suggest that despite the transplantation of the cells in the lung, this treatment method has significant systemic effects. Investigating the transplantation of human MSCs in reducing induced-ALI by use of intravenous oleic acid in a pig model displayed that IL-8 elevation acts as a chemokine for neutrophil and is closely related to the intensity and duration of ALI. Therefore, there was a significant relationship between the neutrophil and the IL-8 concentration but a significant decrease in transcription of NF-κB inflammatory factor [37].

## The effects of MSC therapy on blood gases situation

The results of the analysis of blood gases have an important role in the diagnosis and management of pulmonary capacity, the state of oxygenation and the balance of acid and base. The low level of partial pressure of oxygen (PO2) and subsequent high level of and partial pressure of carbon dioxide (PCO2) in the blood can occur due to the decline in gases exchange resulting from inflammation and obstruction of the airways which causes the hypoventilation and dyspnea. If the low level of PO2 (hypoxia) is prolonged, it can cause contraction of the blood vessels in the lungs and the pulmonary hypertension [84]. The researchers found that the cell therapy with the MSCs has been able to improve the blood PO2 level through the mediators and inflammation reduction. The stem/stromal cells transplantation induces a similar effect with the bronchodilator drugs and has led to a significant increase in arterial blood oxygenation and a the decrease in carbon dioxide level. They found that in the ARDS experimental model in rabbit that had decreased the O2 saturation (SatO2) level, the MSCs transplantation has caused a significant increase in the amount of SatO2 and the arterial blood pressure after 12 h [5] and also, in ventilation-induced lung injury in rat caused a significant increase in the oxygenation of arterial blood [63]. The PCO2 is evaluated for the pulmonary ventilation condition and as an the effective respiratory component in the regulation of blood acidity. In the pulmonary diseases, hypoventilation leads to imprisonment and accumulation of the CO2 in the blood (increase PCO2) and a decrease in the pH (respiratory acidosis). The transplantation of MSCs in the ARDS of rabbit caused deep and fast respiration (hyperventilation) which increased the CO_2_ elimination (decreased PCO_2_) and resulted in an increase in the blood pH [5]. Li et al., also stated that the use of MSCs in inflammation caused by H9N2 avian influenza in mice decreases the lung physiological dysfunction indices and increases the lung capability [52].

## The effects of MSC therapy on edema and inflammations of the lung

Tomography is used to represent the heterogeneous patterns in the pulmonary diseases. The computed tomography scan (CT-scan) can provide valuable diagnostic information in the patients with pulmonary diseases, various stages of disease, improvement, or complications for the clinicians. In the CT-scan studies, increasing the Honsfield unit confirms the replacement of alveolar air with the mucus and inflammatory cells and the mean volume of lung increases as a result of edema and inflammation [85]. Accordingly, the examination of the lung CT-scan at different times post-MSCs therapy in the ARDS model in the rabbit demonstrated a significant decrement in Honsfield unit 1 week after transplantation, that confirmed the effect of the stem/stromal cells in reduction of the pulmonary inflammation and edema [5].

The evaluation of histopathology after the cellular treatment of the pulmonary inflammation in mice revealed a significant decrease in inflammatory exudate and interstitial edema, decline in the thickness of the wall between the alveolar [30, 59] and a significant reduction in the neutrophils of the lung compared with the control group. However, the histopathological study did not reveal significant statistical differences in the pig model [37]. Also, the pathological findings of Li et al. showed that the MSCs-based therapy could help to lessen the lung histopathologic changes, including reduced lung inflammation and the pulmonary vascular permeability, and help in the treatment of H9N2 avian influenza, but the correlation of their data was not statistically significant [52] and Shalaby et al. showed the BM-MSCs protect anatomical structure of the lung in E.coli-induced ALI in mice [67]. Also, the intravenous transplantation of adipose tissue-derived MSCs in the model of endotoxin-induced ALI in mice lead to the least infiltration of inflammatory cells, no apoptosis, and maintained lung architecture [64]. In another study in the ARDS model in rabbit, the absence of calcification or granulation formation and the subacute and chronic granulomatous reactions, the reduction of inflammation and hemorrhage, and the frequent hotspots of ATII cells production after the MSCs transplantation compared to control group was reported. They stated that the lung probably responds well to transplantation of the MSCs due to its anatomical structure and in this regard better blood supply, less connective tissue, less lobulation, the alveolar relationship to each other, having the side ventilation and the ease of access to blood factors (humoral and cellular factors) have a role [5].

## Conclusion

This review revealed that the MSCs play an important role in the repair of the acute lung injuries. So that, these cells prevent the progression of inflammation and edema and improve the clinical signs and the local and systemic inflammatory factors. The released agents by the MSCs play a the crucial role in the repair of injury through the properties of anti-inflammatory, anti-apoptosis, angiogenesis and the immune system modification, and also, the lung damage can be improved by the ability of the MSCs to differentiate to the cells of lung epithelium and endothelial. However, although many advances have been made in the treatment of acute pulmonary diseases using the MSCs, and it is considered a safe and effective treatment method, more pre-clinical trials are needed to find the best cell source, the cell culture and suitable passage, the storage conditions, the cell count, the method of transplantation and cell transfer time. So the present review, along with the complementary reports, can use the MSCs therapy for the treatment of acute lung diseases in the future.

## Data Availability

Authors want to share their data.

## Abbreviations

MSCs: Mesenchymal Stem/Stromal Cells
ARDS: Acute Respiratory Distress Syndrome
PMNs: PolyMorphonuclear Leukocytes
ATII: Alveolar Type II
ATI: Alveolar Type I
BADJ: Bronchoalveolar Duct Junction
COPD: Chronic Obstructive Pulmonary Disease
IPF: Idiopathic Pulmonary Fibrosis
BPD: Bronchopulmonary Dysplasia
CF: Cystic Fibrosis
LPS: Lipopolysacaride
(TNF-α): Tumor Necrosis Factor-α
IDO: Indolearnine 2,3-dioxygenase
IL-2: Interlukine-2
IL-10: Interlukine-10
CCL18: Chemokine (C-C motif) ligand 18
PGE2: Prostaglandin E2
KGF: Keratinocyte Growth Factor
ALI: Acute Lung Injury
E.coli: *Escherichia coli*
BM-MSCs: Bone Marrow Mesenchymal Stem/Stromal Cells
BAL: Bronchoalveolar Lavage
GFP: Green Fluorescent Protein
NETs: Neutrophil Extracellular Traps
IFN-γ: Interfron-γ
IgM: Immunoglobulin M
MIP-2: Macrophage Inhibitory Protein-2
UC-MSCs: Umbilical Cord Mesenchymal Stem/Stromal Cells
NF-κB: Nuclear Factor kappa B
GM-CSF: Granulocyte Macrophage Colony Stimulating Factor
G-CSF: Granulocyte Colony Stimulating Factor
PO2: partial pressure of Oxygen
PCO2: partial pressure of Carbon dioxide
SatO2: O2 Saturation
CT-scan: Computed Tomography scan
